# Open Partial Nephrectomy in the Management of Small Renal Masses

**DOI:** 10.1155/2008/309760

**Published:** 2008-07-15

**Authors:** Ziya Kirkali, A. Erdem Canda

**Affiliations:** ^1^Department of Urology, Faculty of Medicine, Dokuz Eylul University, 35340 Izmir, Turkey; ^2^1st Urology Clinic, Ankara Ataturk Training and Research Hospital, 06520 Ankara, Turkey

## Abstract

*Introduction*. Most of the kidney masses are being detected incidentally with smaller size due to widespread use of imaging modalities leading to increased RCC incidence worldwide with an earlier stage. This article reviews the role of open partial nephrectomy (PN) in the management of small renal masses. *Material and Methods*. Review of the English literature using MEDLINE has been performed between 1963–2008 on small renal masses, partial nephrectomy, kidney cancer, nephron sparing surgery (NSS), radical nephrectomy, laparoscopy, and surgical management. Special emphasis was given on the indications of NSS, oncological outcomes and comparison with open and laparoscopic PN. *Results*. Overall 68 articles including 31 review papers, 35 human clinical papers, 1 book chapter, and 1 animal research study were selected for the purpose of this article and were reviewed by the authors. *Conclusions*. Currently, open NSS still remains as the gold standard surgical treatment modality in patients with small renal masses.

## 1. INTRODUCTION

Renal cell carcinoma (RCC) accounts for 3% of adult solid tumors; and the highest incidence of RCC is detected between 50–70 years of age [1, 2]. Almost 20,000 renal cancer patients are estimated to be detected yearly in the European Union [3]. In the pathogenesis of conventional RCC, mutations leading to inactivation of the von Hippel Lindau (VHL) tumor suppressor gene have been detected in the hereditary and up to 80% of sporadic forms of clear cell RCC. Premalignant lesions in the kidney such as renal intraepithelial neoplasia have been described, which seems to be sharing similar genetical changes with RCC [4, 5]. Independent predictors of survival in patients with RCC are limited. Tumor stage, grade, and patient-performance status are the known prognostic indicators [6].

Currently, most of the kidney masses are being detected incidentally up to 40% with smaller size due to widespread use of imaging modalities such as ultrasound (US), computed tomography (CT), and magnetic resonance imaging (MRI). This leads to increased RCC incidence worldwide [7] with an earlier stage which can be cured by surgery [8].

This paper reviews the role of open partial nephrectomy (PN) in the management of small renal masses particularly focusing on indications, oncological outcomes and comparison with laparoscopic PN.

## 2. MATERIALS AND METHODS

Review of the English literature using MEDLINE has been performed between 1963–2008 on small renal masses, partial nephrectomy, kidney cancer, nephron sparing surgery, radical nephrectomy, indications, outcomes, surgical management, and laparoscopy.

Overall 68 articles including 31 review papers, 35 human clinical papers, 1-book chapter, and 1 animal research study were selected for the purpose of this article and were reviewed by the authors.

## 3. RESULTS AND DISCUSSION

Small renal masses are considered as tumors less than 4 cm in size in the kidney although there is not an established consensus concerning a clear cut-off value for the definition of a “small renal mass.” However, although 4 cm is commonly considered as the size limit for nephron sparing surgery (NSS) in kidney tumors, when technically feasible, partial nephrectomy (PN) should be performed irrespective of tumor size [9].

### 3.1. Radical nephrectomy versus partial nephrectomy

Radical nephrectomy (RN) was first described by Robson in 1963; it has been the standard for the surgical treatment of kidney cancer [10]. Traditionally, RN can be regarded as the optimal technique with long-term cancer control in kidney cancer [11]. Five-year cancer specific survival for patients with organ-confined disease is over 90% after surgery alone. Since 15–25% of incidentally detected tumours are benign, removing the whole kidney for a small benign lesion is not logical [12]. Current indications of open RN can be summarized as large tumor size which is not suitable for NSS or for laparoscopy, locally advanced diseases, existence of complicated tumor thrombus with vena cava extension, and presence of other concomitant diseases such as renal artery stenosis or single-organ metastases necessitating open surgery [13]. Due to improved technology regarding radiologic imaging modalities and their frequent use, currently most of the kidney tumors are detected incidentally with smaller tumor size and are associated with less lymph node and adrenal gland involvement [14]. Therefore, there is a tendency to perform NSS rather than RN in suitable kidney tumors particularly with recent improvements in surgical techniques.

PN was first performed by Czerny in 1887 [15] and Vermooten described indications of conservative surgery in kidney tumors in 1950 [16]. The goal of NSS is to preserve as much normal renal parenchyma as possible and meticulous cancer control with negative surgical margins and no local recurrence in the follow-up [17]. Multiple studies in the last decade have established the safety and efficacy of PN for selected cases with small renal tumors [11, 18, 19]. Such considerations have led to expanding the indications of PN to include centrally located tumors and larger tumors up to 7 cm [18, 19].

### 3.2. Indications of open partial nephrectomy

The TNM 1997 classification considers tumor size of 4 cm as cut-off value in order to classify stage T1 tumors as T1a (≤4 cm) and T1b (4–7 cm) [20]. Excellent outcomes regarding tumors less than 4 cm in size treated with NSS have an important impact in this staging. Current indications for open PN are summarized on Table 1. In elective setting when contralateral kidney is normal, NSS should be attempted whenever feasible irrespective of the status of the contralateral kidney.

### 3.3. Surgical technique and complications

**Figure 1:**
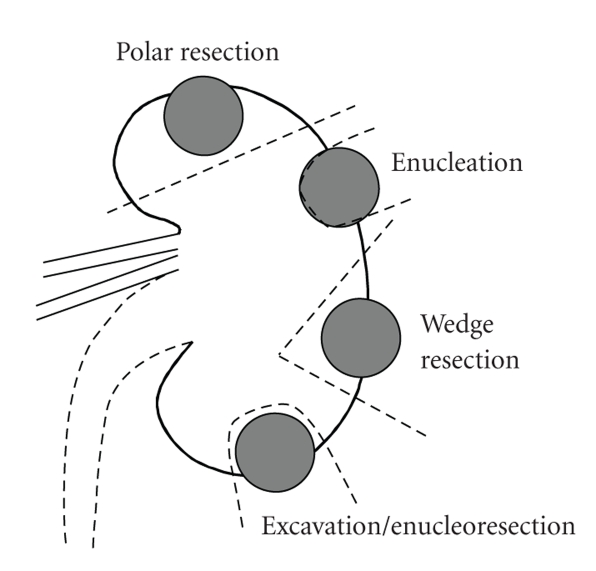
Techniques used in open NSS. NSS: Nephron sparing surgery.

We prefer a flank incision and a lumbar extraperitoneal approach. Kidney is mobilized completely and explored for satellite lesions. If necessary, intraoperative ultrasound can be used. Renal vessels are controlled by using vascular clamps, vascular tape, or by the surgeon's fingers. In difficult cases the artery and the vein are clamped, and ice slush should be applied in order to cool down the kidney. Scalpel, laser, ultrasonic aspirator, water jet, cautery, blunt dissection, or combinations of these can be used to cut the renal parenchyma in order to remove the tumor with surrounding few millimeters of healthy parenchyma and together with the covering perirenal fat. In case of any suspicion in terms of surgical margins, further resection can be performed. Frozen section examination of the tumor bed is usually not helpful. Bleeders are coagulated or sutured and collecting system is closed by absorbable sutures in a water-tight manner if it has been opened. Peroperative hydration and diuresis by mannitol infusion are very helpful. Absorbable sutures for approximation of the renal parenchyma in a “suture of eight” or “Z” sutures fashion are useful. Perirenal fat or omentum can be used in order to close the defect. A drain is placed in the retroperitoneum and wound is closed. Because tumor cells might remain in the residual kidney after resection, enucleation is usually discouraged (Figure 1) [21].

Renal failure, post operative hemorrhage, urine leak, and urinary fistula are the most frequently seen complications after open NSS [21, 22]. Recently, Van Poppel et al. compared the complications of elective open NSS surgery and RN for low-stage, incidentally detected, solitary, small (≤5 cm) RCCs in a prospective study in the presence of a normal contralateral kidney (Table 2). They concluded that NSS can be performed safely in this patient group with slightly higher complication rates than after RN [23].

### 3.4. Outcomes of open partial nephrectomy

Similar cancer-specific survival rates and oncologic outcomes have been detected in patients with small (<4 cm) renal masses who underwent RN or NSS. Therefore, currently NSS is considered as the treatment of choice in these patients (T1a tumors) [9, 24–29] (Table 3).

Ten-year oncological and functional follow-up data revealed almost 100% survival, especially in patients with renal tumors less than 4 cm in size [34–38] with PN. Therefore, open NSS is currently accepted as the gold standard treatment modality for patients with small, exophytic, easily resectable renal masses [19, 34]. In a series of 435 patients who underwent NSS for a tumor size between 2.6–4.0 cm, local recurrence was detected in 3 patients (0.7%) with a mean follow-up of 31–76 months, which is ten times lower than the rate for NSS performed for an absolute indication [39]. Local recurrence has been reported to be between 0–12% in NSS which is related with multifocal disease or insufficient resection of the tumor [40]. Local recurrence is expected to be more frequent locally advanced disease [41].

Presence of preneoplastic lesions such as renal intraepithelial neoplasia in the residual kidney might be a factor for the occurrence of local recurrence [4]. However, recurrence due to insufficient resection could be prevented by proper surgical technique [42]. Multifocal tumors can be detected both in large- and small-sized tumors [4]. Although there is a possibility of presence of multifocality and premalignant lesions, local recurrence rate is quite low in well selected cases after NSS [43].

### 3.5. Preoperative kidney biopsy

It has been demonstrated that almost a quarter of all small renal masses (<4 cm) are benign lesions like angiomyolipoma, oncocytoma, or metanephric adenoma. Preoperative diagnosis of these lesions is difficult despite latest advances in imaging techniques [27, 40, 44, 45]. Therefore, performing RN would be unnecessary for these benign lesions. One may possibly think of diagnosing these lesions preoperatively by kidney biopsy. Currently, the role of renal biopsy in diagnosing these lesions is controversial. Although preoperative fine needle aspiration biopsy can be performed for diagnosis, its sensitivity is low, has complication risks such as bleeding and tumor seeding, might give false positive and false negative results, and finally it needs an experienced cytopathologist particularly subspecialized in kidney and RCC [17, 46]. We suggest kidney biopsy particularly in those patients where renal lymphoma or metastatic involvement of the kidney is suspected [47].

### 3.6. Optimal margins in open NSS and significance of performing tumor bed biopsies

For tumors smaller than 4 cm, the local/ipsilateral renal recurrence rate has been reported to range between 1.5 and 4% in open NSS series [34, 36]. In the past, a 1 cm normal parenchyma was suggested as a safety margin in NSS but controversy exists concerning the optimal margin width [48]. Intraoperative biopsy and frozen-section examination of the tumor bed is suggested in order to rule out residual tumor in the kidney [49]. However, false-positive and false-negative results can be obtained due to freezing artifacts and difficulty in distinguishing cancer cells from normal cells [50] which might also lead to unnecessary resections or even RN [51]. It has been shown that more than 30% of small renal tumors (≤4 cm) did not have an intact pseudocapsule; and cancer cells might be detected beyond the pseudocapsule reaching up to 0–5 mm [52] therefore, an amount of normal kidney tissue surrounding the tumor is suggested to be included with PN in order to prevent incomplete resection [53]. This amount has been recommended to be at least 5 mm in NSS by some authors [52], whereas others suggest a normal tissue safety margin of ≥1 mm to be removed [54]. In conclusion, the margin status rather than size seems to be important in NSS and 1 mm of normal parenchyma around the tumor seems to be enough.

It is known that RCC has a 1–5% recurrence rate in the contralateral kidney particularly in surgical margin positive patients and patients with multifocal tumors which support NSS in this patient group [55, 56]. Several authors suggest intraoperative use of ultrasonography to rule out multifocal disease, and to clearly define tumor extent [57, 58]. Coagulation of the tumor bed in addition to biopsies is recommended when tumor enucleation is performed [59].

### 3.7. Open versus laparoscopic partial nephrectomy

In the recent years, laparoscopy has gained popularity and emerged as an alternative to open PN in the treatment of renal masses [58]. Technical advances have enabled laparoscopists to duplicate the techniques used during open PN, including vascular control, hemostasis, and repair of the pelvicalyceal system [58, 60]. Promising postoperative and intermediate-term oncological outcomes have been reported with laparoscopic partial nephrectomy (LPN) [57, 61, 62].

Recently, Lane BR and Gill IS reported their 5-year outcomes in LPN including 58 patients which are comparable to those of open NSS. At a median follow-up of 5.7 years, no distant recurrence and a single local recurrence (2.7%) were detected. Overall and cancer-specific survival was 86% and 100%, respectively, at 5 years [62]. Moinzadeh et al. also reported oncological results in 100 patients with a minimum follow-up of 3 years. Overall survival was 86%, and a cancer-specific survival was 100% [61].

Although LPN seems to be a promising and attractive surgical approach in the management of renal masses, there are still some problems for LPN. Bleeding and hemostasis, prolonged warm ischemia, longer operative time, increased intraoperative and renal/urological major complication rates are considered as current problems associated with LPN. Hemostasis and ischemia time is the most challenging steps in LPN [60]. Bleeding during LPN is an important problem for the surgeon although improved surgical techniques and skills together with the use of new hemostatic sealants such as fibrin glue-coated collagen patch which contains purely human coagulation factor components can be helpful in order to overcome this problem [63–65].

There are several studies investigating the impact of the warm ischemia time on renal functions and as a widely accepted guideline for clinical practice, warm renal ischemia period exceeding 30 minutes is not recommended [66]. Furthermore, it is technically very demanding and time consuming to produce cold ischemia during laparoscopic surgery [66]. Preservation of maximum functional kidney tissue is one of the goals in PN, however, longer warm ischemia times have been reported with LPN [33] compared to open NSS [30, 37].

The operating time seems to be decreased for LPN in the most experienced centers [30] however the learning curve is not short and technical feasibility of an operation does not always necessarily mean that it can be performed in common practice. This is still a major issue when health care costs to society are concerned [12].

Significantly increased major complication rates have been reported with LPN compared to open NSS by experienced authors [30, 37, 67]. However, for peripherally located, small, and exophytic renal masses, we expect these complications to be lower.

The risk of tumor spillage is also a theoretical problem in LPN [60]. However, tumor spillage has been reported at port sites in patients undergoing laparoscopic nephrectomy and nephroureterectomy due to tumor [68].

Decreased analgesic requirement, decreased hospital stay, shortened convalescence, and improved cosmetics are considered as the main advantages of LPN. The length of stay for patients undergoing LPN in large series from Europe [31, 32] is ranging from 6 to 9 days whereas the average length of stay in the United States is between 2 and 4 days [30, 33]. Gill et al. reported their results comparing open versus laparoscopic PN (Table 4) [30]. Characteristics of some selected series of LPN are summarized on Table 5 [31–33].

The follow-up after LPN is shorter compared to open NSS concerning oncologic outcomes. LPN has a long learning curve and requires high-level laparoscopic skills and experience. Long-term data indicate that NSS is safe and oncologically effective in small renal masses <4 cm in size. Until the problems with LPN are overcome, high complication rates are lowered and longer oncological follow-up data are available, open NSS will be the standard treatment for the surgical management of kidney tumors [12].

## 4. CONCLUSIONS

Due to widespread use of radiologic imaging modalities, most of the kidney tumors are being detected incidentally with smaller size and earlier stage. Similar oncologic outcomes have been detected in patients with small (<4 cm) renal masses who underwent RN or NSS. Currently, NSS is considered as the treatment of choice in patients with kidney tumors when technically feasible irrespective of tumor size. In last few years laparoscopy has gained popularity and emerged as an alternative to open PN particularly in the surgical management of small renal masses. However, complication rates are higher and oncological follow-up data is shorter compared to open PN therefore, NSS still remains as the gold standard surgical treatment modality in patients with small renal masses.

## Figures and Tables

**Table 1:** Open partial nephrectomy indications (table adapted from 12).

**I. Absolute Indications**
a. Tumors in a solitary kidney
b. Bilateral synchronous renal masses
c. Severe renal insufficiency

**II. Relative Indications**
a. Presence of pre-existing renal disease in the contralateral kidney
*1. Nephrolithiasis*
*2. Recurrent pyelonephritis*
*3. Mild-moderate renal insufficiency*
*4. Ureteropelvic junction obstruction*
*5. Vesicoureteral reflux*
b. Presence of diseases predisposing to renal insufficiency
*1. Diabetes*
*2. Hypertension*
c. Patients with known multifocal disease or underlying genetic syndromes
*1. Papillary RCC*
*2. Von Hippel-Lindau disease*

**Table 2:** Complications of open NSS and RN [23].

	NSS (%), (*n* = 268)	RN (%), (*n* = 273)
Rate of severe haemorrhage	3.1	1.2
Pleural damage	11.5	9.3
Spleen damage	0.4	0.4
Postoperative CT abnormalities	5.8	2.0
Urinary fistula development	4.4	—
Reoperation for complications	4.4	2.4

**Perioperative blood loss was slightly higher after RN (*p* > .05). NSS: Nephron sparing surgery, RN: Radical nephrectomy, CT: Computed tomography.

**Table 3:** Selected published series including patients who underwent open NSS or RN for renal masses due to their tumor size (table modified from [14]).

Author	Reference	Year	*N*		Local recurrence (%)		5-year dfs (mos)	

			Tumor size <4 cm in size*		

Hafez et al.	[26]	1999	310		0.6		96	
Lee et al.	[27]	2000	79		0		100	
McKiernan et al.	[24]	2002	117		1.2		100	
Patard et al.	[18]	2004	314		0.8		98	

			Tumor size >4 cm in size**		

Hafez et al.	[26]	1999	175		0.8		86	
Patard et al.	[18]	2004	65		3.6		94	
Leibovich et al.	[28]	2004	91		5.4		98	
Becker et al.	[29]	2006	69		5.8		100	

Selected series comparing outcomes of patients underwent NSS or RN for renal masses*							

			RN	NSS	RN	NSS	RN	NSS

Patard et al.	[18]	2004	1075	379	99	99	97	98
Lee et al.	[27]	2000	183	79	100	100	96	96
Leibovich et al.	[28]	2004	841	91	98	95	86	98
McKiernan et al.	[24]	2002	173	117	99	96	100	100

*Median follow-up is >25 months for all studies. **Median follow-up is >47 months for all studies. *N*: Number of patients, dfs: disease-free survival, FU: Follow-up, mos: months.

**Table 4:** Comparison of laparoscopic versus open PN in patients with a solitary renal tumor of 7 cm or less in size (table modified from [30]).

		Laparoscopic PN (*n* = 100)	Open PN (*n* = 100)	*p*
Complications:	Major intraoperative	5%	0%	.02
	Renal/urological	11%	2%	.01
Median surgical time (hours)		3	3.9	<.001
Blood loss (mL)		125	250	<.001
Mean warm ischemia time (minutes)		27.8	7.5	<.001
Median analgesic requirement (morphine sulfate equivalents, mg)		20.2	252.5	<.001
Hospital stay (days)		2	5	<.001
Average convalescence (weeks)		4	6	<.001
Median preoperative serum creatinine (mg/dL)		1.0	1.0	.52
Median postoperative serum creatinine (mg/dL)		1.1	1.2	.65

PN: Partial nephrectomy.

**Table 5:** Comparison of selected published series related with LPN. Min: Minutes.

Authors	Crepel et al.	Häcker et al.	Haber et al.
Reference	[31]	[32]	[33]
Center	Mutlicenter study	Elisabethinen hospital	Cleveland clinic
	France	Austria	Ohio, USA
Year	2007	2007	2006
Number of patients	91	25	>500
Tumor size (cm)	2.7	2.6	2.9
Route	Transperitoneal	Transperitoneal	Transperitoneal
	retroperitoneal		retroperitoneal
Warm ischemia time (min)	35	29	32
Mean operating time	163 min	212 min	Transperitoneal: 3.5 h
			Retroperitoneal: 2.9 h
Complication rate (%)	17.6	8	36 and 16
Mean blood loss (mL)	363	177.4	150 versus 100 and 231
Transfusion rate (%)	6.6	4	Not reported
Hospital stay (days)	9.1	8.3	Transperitoneal: 2.9
			Retroperitoneal: 2.2

LPN: Laparoscopic partial nephrectomy.
